# Major channels involved in neuropsychiatric disorders and therapeutic perspectives

**DOI:** 10.3389/fgene.2013.00076

**Published:** 2013-05-07

**Authors:** Paola Imbrici, Diana Conte Camerino, Domenico Tricarico

**Affiliations:** Section of Pharmacology, Department of Pharmacy - Drug Science, University of BariBari, Italy

**Keywords:** ion channels, bipolar disorders, schizophrenia, autism, ion channels blockers, ion channel openers

## Abstract

Voltage-gated ion channels are important mediators of physiological functions in the central nervous system. The cyclic activation of these channels influences neurotransmitter release, neuron excitability, gene transcription, and plasticity, providing distinct brain areas with unique physiological and pharmacological response. A growing body of data has implicated ion channels in the susceptibility or pathogenesis of psychiatric diseases. Indeed, population studies support the association of polymorphisms in calcium and potassium channels with the genetic risk for bipolar disorders (BPDs) or schizophrenia. Moreover, point mutations in calcium, sodium, and potassium channel genes have been identified in some childhood developmental disorders. Finally, antibodies against potassium channel complexes occur in a series of autoimmune psychiatric diseases. Here we report recent studies assessing the role of calcium, sodium, and potassium channels in BPD, schizophrenia, and autism spectrum disorders, and briefly summarize promising pharmacological strategies targeted on ion channels for the therapy of mental illness and related genetic tests.

## INTRODUCTION

Important advances have been made in the diagnosis and treatment of psychiatric disorders. A growing body of data, indeed, supports the view that these disorders arise through the interplay of genetic ( Askland et al., 2012; Devlin and Scherer, 2012; Mulle, 2012; Sullivan et al., 2012) and environmental risk factors ( Karmiloff-Smith et al., 2012) and result from structural and functional impairments in various brain regions such as thalamus, amygdala, mid-brain, and the prefrontal cortex ( Gargus, 2006; Squintani et al., 2010; O’Donnell, 2012; Offord, 2012; Piper et al., 2012; Price and Drevets, 2012; Stachowiak et al., 2013). Moreover, specific pattern of brain activity have been correlated to the clinical phenotype of some mental disorders ( Bigos et al., 2010). Voltage-gated ion channels and inward rectifier potassium channels are widely and selectively distributed in the brain, both in neurons and astrocytes, often forming macromolecular complexes with associated proteins or auxiliary subunits within distinct subcellular compartments. The alternating activation of different ionic currents sets the resting membrane potential, generates the action potential, and regulates the neuronal firing frequency and neurotransmitter release, thus providing neuronal cells with specific electrical identity ( Hibino et al., 2010; Catterall, 2012; Jan and Jan, 2012). Since the early 1990s, ion channel genes have been mapped on human chromosomes and mutations in these genes have been subsequently identified. Several neurological diseases such as epilepsy, episodic ataxias, migraine, due to abnormal functioning of brain electrical circuits have been attributed to ion channel mutations ( Gargus, 2009; Kullmann, 2010; Imbrici et al., 2011). Therefore, it is not surprising that alterations in ion channels activity in the relevant brain areas may be also claimed in the etiopathology of psychiatric disorders ( Gargus, 2006, 2009; Liao and Soong, 2010; Askland et al., 2012; O’Roak et al., 2012).

This review focuses on the emerging pathological role of calcium, sodium, and potassium channels in bipolar disorder (BPD), schizophrenia, and autism spectrum disorders (ASDs; **Table 1**). Some neuropsychiatric syndromes of autoimmune origin will also be discussed. The involvement of neurotransmitter operated ion channels, like nicotinic, glycine, GABA, and glutamate receptors, is also well established in schizophrenia and BPD, but is beyond the scope of this paper (for reviews, see Gonzalez-Burgos et al., 2011; Benes, 2012; Egerton and Stone, 2012; Fountoulakis, 2012; Javitt, 2012). The contribution of ion channels to the etiology of neuropsychiatric disorders can be variable. Ion channels can be the unique cause of the disease, as occurs in Timothy syndrome (TS), an autosomal dominant disease caused by calcium channel mutations ( Gargus, 2009). In most cases, however, the association of ion channels genetic variants with major psychiatric disorders is far more complicated, due to the complex genetic architecture of these disorders. Until recently in fact, information about the potential association between psychiatric diseases and genetic predisposing factors came from genome-wide association studies (GWAS) based on a common disease/common variant (CD/CV) model, where the disease resulted from the inheritance in any individual, of a combination of several common variants, each contributing with small effect ( Mitchell and Porteous, 2011). However, the latest findings demonstrate that major psychiatric disorders are also caused by rare and often *de novo* mutations of large effect ( Mulle, 2012; O’Roak et al., 2012). Furthermore, ion channels mutations are often found in patients where epilepsy occurs together with psychiatric symptoms, suggesting fundamental overlapping etiology of apparently clinically distinct disorders ( Derry et al., 2008; Hermann et al., 2008; Sicca et al., 2011). Furthermore, disease-modifying variants in each individual can account for the phenotypic variability typical of mental illness ( Mitchell, 2011). For some of these disorders a genetic test is available (**Table 2**). The drugability of ion channels supports research efforts to clarify the dynamics of ion channels contribution in functional brain disorders, and to identify compounds with therapeutic potential.

**Table 1:** Ion channels involved in main neuropsychiatric disorders.

**Ion channel type/disease**	**Gene**	**Protein**	**Chromosome**	**Pharmacological perspectives**	**Reference**
**Calcium**
Bipolar disorder, schizophrenia, autism spectrum disorders, Timothy syndrome	*CACNA1C*	Cav1.2 α subunit	12p13.3	Cav channel blockers (verapamil, nicardipine) as antipsychotics;α_2_β_4_ ligands (pregabalin) as antidepressants	Gargus (2009), International Schizophrenia Consortium (2009), Liao and Soong (2010), Lee et al. (2012); Valente et al. (2012)
	*CACNG2,4,5*	Calcium channel γ subunits	22q13.1, 17q24		Wilson et al. (2006), Curtis et al. (2011)
	*CACNA2D4*	Calcium channel α_2_β_4_ subunit	12p13.3		Van Den Bossche et al. (2012)
**Sodium**
Autism spectrum disorders/epilepsy, Dravet syndrome	*SCN1A*	Nav1.1 α subunit	2q24.3	Nav channel blockers (lamotrigine, phenytoin, carbamazepine) as mood stabilizers and antidepressants; antidepressants (fluoxetine, sertraline) block Nav channels	Aldana and Sitges (2012); Bender et al. (2012), O’Roak et al. (2012); Sanders et al. (2012), Perucca and Mula (2013)
	*SCN2A*	Nav1.2α subunit	2q24.3		Chen et al. (2010), Bartnik et al. (2011), Psychiatric GWAS Consortium Bipolar Disorder Working Group (2011), Sanders et al. (2012)
	*SCN3A*	Nav1.3 α subunit	2q24		Davidsson et al. (2008); Chen et al. (2010)
**Potassium**
Bipolar disorder, schizophrenia, autism spectrum disorders, autoimmune neuropsychiatric disorder	*KCN1A*	Kv1.1	12p13.32	Kv1.1 “disinactivators” as potential anticonvulsants and in neuropathic pain	Lu et al. (2008), Illingworth et al. (2011), Somers et al. (2011)
	*KCNQ3*	Kv7.3	8q24	KCNQ openers (retigabine) as anticonvulsant and antipsychotic drugs; KCNQ inhibitors (linopirdine) for learning disabilities	Zhang et al. (2010a), Psychiatric GWAS Consortium Bipolar DisorderWorking Group (2011); Kristensen et al. (2012)
	*KCNH2*	HERG1, Kv11.1	7q36.1		Atalar et al. (2010)
	*KCNJ3*	Kir3.1	2q24.1		Yamada et al. (2012)
	*KCNJ10*	Kir4.1	1q23.2	Selective serotonin reuptake inhibitors (fluoxetine, escitalopram) and tricyclic antidepressants (nortriptyline) block Kir channels	Ohno et al. (2007); Sala-Rabanal et al. (2010), Williams et al. (2010), Sicca et al. (2011)
	*KCNJ8/KCNJ11*	Kir6.1/Kir6.2	12p11.23/11p15.1	KATP openers (diazoxide, iptakalim) as antipsychotics	Sun et al. (2010); Mele et al. (2012)
	*KCNN3*	SKCa3 (SK3)	1q21.3	SK3 openers for learning difficulties and schizophrenia	Gargus (2006), Grube et al. (2011)
	*KCNMA1*	KCa1.1	10q22.3		Laumonnier et al. (2006)
	*KCNK9*	TASK3	8q24.3		Barel et al. (2008)

**Table 2:** Genetic tests for some neuropsychiatric disorders associated to ion channels dysfunctions (http://www.orpha.net).

**Disease**	**Gene (protein)**	**Chromosome**
Timothy syndrome	*CACNAJC* (Cav1.2)	12p13.3
SeSAME syndrome	*KCNJ10* (Kir4.1)	1q23.2
Birk Barel mental retardation dysmorphism syndrome	*KCNK9* (TASK3)	8q24.3
Dravet syndrome and epileptic encephalopathy early infantile	*SCN1A* (Nav1.1)	2q24.3
Epileptic encephalopathy, early infantile type	*SCN2A* (Nav1.2)	2q24.3
Epileptic encephalopathy, early infantile type 13	*SCN8A* (Nav1.8)	12q13.13
Epileptic encephalopathy, early infantile type 7	*KCNQ2* (KTQ2)	20q13.33

## GENERAL FEATURES OF ION CHANNELS

Voltage-gated ion channels are made up of one or more pore-forming subunits (generally referred to as α subunits), and variable numbers of accessory subunits (often denoted β, γ, etc.). The α subunit is the ion-conducting pore determining the ion selectivity and the voltage-sensing functions of the channel. However, the biophysical properties of native channels isolated from the brain are determined by the presence of cytoplasmic or extracellular modulatory auxiliary subunits ( Jan and Jan, 2012; Weiss and Zamponi, 2012). The α subunits of the voltage-gated potassium, sodium, and calcium channels are all evolutionary related and share a similar structure ( Catterall, 2011; Eijkelkamp et al., 2012; Zakon, 2012).

### VOLTAGE-GATED CALCIUM CHANNELS

Voltage-gated calcium channels (Cav channels) mediate calcium entry into a wide variety of electrically excitable cells, including cardiac and skeletal muscle cells, neurons, endocrine and sensory cells, thereby controlling numerous physiological processes. Ten genes named *CACNA1A-S* encode the pore-channel Cav subunits that generate the L-types (Cav1), the neuronal P/Q-, N-, and R-types (Cav2), and the T-type (Cav3). Cav1 and Cav2 subunits are high voltage-activated channels, which also include regulatory subunits β, α_2_δ, and γ. Among calcium channels, L-type Cav1.2 channel is widely distributed in the brain in the somatodendritic area of neurons, in cardiac myocytes, lymphocytes, pancreatic β-cells. Interestingly, there is increasing information for an association of Cav1.2 with psychiatric disorders ( Ferreira et al., 2008; Sklar et al., 2008).

### VOLTAGE-GATED SODIUM CHANNELS

Voltage-gated sodium channels (Nav channels) are essential for generation and propagation of signals in electrically excitable tissues. Three sodium channel genes encoding distinct α-subunit isoforms *SCN1A*, *SCN2A*, *SCN3A* are highly expressed in neurons and glia throughout the central nervous system (CNS) and peripheral nervous system. Mutations in genes encoding Nav channels are significant factors in the etiology of neurological diseases and psychiatric disorders, including various types of idiopathic epilepsy, ataxia, and sensitivity to pain. The *SCN1A*, *SCN2A*, *SCN3A*, *SCN7A*, and *SCN9A* genes form a 1.4-Mb SCN cluster on chromosome 2q24.3 ( Catterall, 2012).

### POTASSIUM CHANNELS

Potassium channels set the resting membrane potential, repolarize neurons following action potentials, and also mediate some forms of subthreshold signaling. K^+^ channels are classified on the basis of the primary amino acid sequence of the pore-containing α-subunit into three major families: (1) voltage-gated K^+^ channels (Kv) containing six or seven transmembrane regions with a single pore, including also KCNQ, human ether-a-go-go-related gene (hERG), eag, and Ca^2^^+^-activated K^+^ channels; (2) inward rectifiers (Kir) containing only two transmembrane regions and a single pore; and (3) two-pore tandem K^+^ channels containing four transmembrane segments with two pores ( Maljevic and Lerche, 2012). Among neuronal Kv channels, the low voltage-activated Kv1.1 and Kv1.2 are expressed mainly in the cerebellum, hippocampus, and thalamus. These channels, located in the axons of neurons, do not affect the first action potential but increase the action potential threshold and guarantee the correct propagation of electrical signals ( Jan and Jan, 2012). Kv1.1 co-assembles with Kv1.4 at the hippocampal mossy fiber boutons, where they mediate neuronal activity-dependent processes involved in synaptic plasticity ( Imbrici et al., 2007). The *KCNQ2–5* channels are expressed in different combinations in neurons. They mediate the M-currents inhibited by acetylcholine (ACh) through the muscarinic receptors involved in the ACh-dependent postsynaptic depolarization. M-current plays a critical role in thalamic sensory pathways in attention, perception, and memory. It remains one of the most promising ion channel disease candidate gene and it maps to a locus on chromosome 6q14, a region repeatedly suggested containing an attention-deficit/hyperactivity disorder (ADHD) locus, a schizophrenia locus, and bipolar disease susceptibility alleles ( Gargus, 2006). HERG channels form a particular subfamily of voltage-gated potassium channels known as H or Kv11. HERG1 (also referred as *KCNH2* or Kv11.1) plays an important role in the repolarization of the cellular membrane potential of excitable cells, as cardiac, neuronal, and smooth muscle cells ( Maljevic and Lerche, 2012).

Since the cloning of the first inward rectifiers, new members of this family have been identified, including the Kir4.1–2 (*KCNJ10,15*), Kir5.1 (*KCNJ16*), the G protein-coupled (GIRK) Kir3.1–4 (*KCNJ6,5,9,3*), and the ATP-sensitive (KATP) Kir6.1–2 (*KCNJ8,11*). These channels play pivotal roles in many organs including brain, ear, and retina. Kir4.1 subunit is widely expressed in kidney and brain where it can form homotetrameric channel or heterotetramers with Kir5.1 (*KCNJ16*), with distinct physiological properties ( Hibino et al., 2010). GIRKs are widely distributed in the brain and play an important role in regulating neural excitability through the activation of various G protein-coupled receptors ( Hibino et al., 2010). ATP-sensitive K^+^ (KATP) channels are octameric complexes of the pore-forming Kir6.1–2 subunits and of regulatory sulfonylurea receptor (SUR) subunits belonging to ATP-binding cassette protein superfamily. KATP channels couple metabolism to the electrical activity of the cells. Indeed, several studies have shown that they can be associated with glycolytic proteins including pumps and enzymes such as pyruvate kinase (PK), to form macromolecular complexes that contribute to channels regulation ( Dhar-Chowdhury et al., 2005; Mele et al., 2012).

## NEUROPSYCHIATRIC DISORDERS ASSOCIATED TO ION CHANNELS DYSFUNCTIONS

### BIPOLAR DISORDER

Bipolar disorder is a common, chronic and recurring medical disorder characterized by episodes of mania, that is extremely elevated mood, increase in motor activity, racing thought patterns, impaired judgment, decreased sleep and sometime psychosis, and depression ( Offord, 2012). The prevalence of the disease is around 1% of the population, though, due to the problem of misdiagnosis, the prevalence may be higher ( Offord, 2012). Increasing evidence suggests that BPD stems from structural and functional impairments in various brain regions, mainly in corticolimbic structures linked with disrupted emotional and executive functioning ( Cousins and Grunze, 2012; Price and Drevets, 2012). In addition, mood disorders, particularly BPD, are associated with a disruption in mechanisms and factors that govern cell survival and neural plasticity ( Rogawski and Loscher, 2004). Several research groups have performed independent GWAS of BPD supporting the view that ion channels dysfunctions may also contribute to the genetic etiology of this disease.

#### Calcium channels involvement

The strong association of calcium channels to BPD came out from the analysis of published GWAS data, concluding that the Cav1.2 gene (*CACNA1C*) is the most relevant susceptibility locus associated with BPD. Indeed, distinct studies, including the Wellcome Trust Case Control Consortium, STEP-UCL, STEP-BD, and ED-DUB-STEP2, identified one single-nucleotide polymorphism (SNP), rs1006737, located in the third intron of the Cav1.2 gene ( Ferreira et al., 2008; Sklar et al., 2008; Liao and Soong, 2010; Psychiatric GWAS Consortium Bipolar Disorder Working Group, 2011). Furthermore, BPD was also associated with SNP rs10994336 in ankyrin 3 gene (*ANK3*; Ferreira et al., 2008) which encodes a family of proteins that was thought to be involved in the assembly of Nav channels and proper functioning at the nodes of Ranvier ( Poliak and Peles, 2003). One related study by Curtis et al. (2011) also identified a SNP, rs17645023, between the *CACNG5* and *CACNG4* genes in patients with BPD. These latter genes code for two γ subunits of neuronal Cav channel. In addition, Wilson et al. (2006) screened the postmortem brain DNA of 35 BPD cases and reported DNA copy number variations (CNV) in several loci including *GLuR7* (encoding a kainate receptor subunit), *AKAP5* (encoding a member of the A kinase anchor protein), and *CACNG2* (encoding a Cav channel γ subunit). In another study, a total of 709 bipolar patients, 645 patients with schizophrenia, 189 patients with schizoaffective disorder, and 1,470 control individuals were screened using the multiplex amplicon quantification method. A rare, partial deletion of 35.7 kb was found in *CACNA2D4* (encoding the Cav channel α_2_δ_4_ subunit), in two unrelated late onset bipolar patients and in one control individual ( Van Den Bossche et al., 2012). The functional correlation of these genetic variants to the pathogenesis of BPD is largely unknown; some hypotheses can be made, though. First, *CACNA1C* and *ANK3* genes are involved in glutamatergic signaling which is integral to normal neuronal function ( Wilson et al., 2006) and was previously found to play a role in BPD etiology ( Liao and Soong, 2010). Second, the most effective drug to treat BPD, lithium, was reported to down-regulate both ANK3 and Cav1.2 channel in the mouse brain ( Lee et al., 2012). Third, the gamma subunits stabilize the calcium channel in an inactivated state and regulate the trafficking and gating properties of AMPA-selective glutamate receptors. Finally, the α_2_δ_4_ subunit encoded by *CACNA2D4* associates with the Cav1.2 α subunit and doubles calcium influx. The *CACNA1C* gene is also localized in proximity of the *CACNA2D4* gene on chromosome 12, as both genes are only 135 kb apart.

An attempt to give functional relevance to genetic data and to shed light into neural mechanisms of genetic risk was proposed by Bigos et al. (2010). They used functional MRI to find a correlation between the occurrence of *CACNA1C* SNPs and specific patterns of brain activity characteristic of patients affected by mental illness ( Bigos et al., 2010). Indeed, they found that healthy individuals carrying the SNP rs1006737 showed increased hippocampal activity in response to emotional stimuli, a pattern that has been associated with the risk for BPD; the same individuals also showed increased prefrontal activity during working memory tasks, a clinical feature typical of schizophrenia. Moreover, this study demonstrated that the molecular mechanism of genetic risk appears to relate at least in part to regulation of gene expression. Indeed, carriers of the risk SNP had increased levels of *CACNA1C* mRNA in the human dorsolateral prefrontal cortex measured from postmortem brain samples. Calcium channels are involved in various aspects of neuronal maturity during development and throughout adulthood ( Gargus, 2009; Liao and Soong, 2010); therefore alterations in gene expression may also affect the functional activity of brain circuitries implicated in both mental conditions. This study also suggests that it may be worth considering genotype or brain imaging based phenotypes as individual predictors of response to psychiatric agents in clinical trials ( Liao and Soong, 2010).

#### Potassium channels involvement

Potassium channels have been proposed to play a role in mechanisms of neural plasticity which are altered in various psychiatric disorders, especially in hippocampus ( Askland, 2006; Fontan-Lozano et al., 2011; Shah et al., 2011; Kristensen et al., 2012). Multiple linkage and association studies have suggested chromosome 8q24 as a promising candidate region for BPD ( Park et al., 2004; Zandi et al., 2008). Zhang et al. (2010a) performed a detailed association analysis on 2,756 SNP markers in the chromosome 8q24 region of 3,512 individuals from 737 families. Their results showed suggestive evidence of association of BPD with loci near *KCNQ3* gene, encoding the voltage-gated potassium channel Kv7.3. Kiselycznyk et al. (2012), instead, excluded the involvement of Kir4.2 in anxiety or depression by analyzing the neurological functions of *KCNJ15* knock-out mice on a battery of behaviors including those related to anxiety and depression, and on plasticity-related learning tasks.

### SCHIZOPHRENIA

Schizophrenia is a serious mental disorder affecting around 1% of the general population worldwide ( Askland et al., 2012; Mulle, 2012; Sullivan et al., 2012). Symptoms of schizophrenia include dysfunction of executive functioning (symptoms of cognitive disorganization), social responsiveness (negative symptoms, or asociality), and sensory-perceptual integration (classical positive symptoms, including hallucinations and delusions; Askland et al., 2012). Several lines of evidence increasingly suggest that schizophrenia is a disorder of brain development and plasticity, where the activity and excitability of hippocampus, substantia nigra, ventral tegmental area, and prefrontal cortex are strongly altered ( Eichhammer et al., 2004; Oxley et al., 2004; O’Donnell, 2012; Silveira et al., 2012; Stachowiak et al., 2013). GABA and glutamate neurotransmission emerged as critical elements in schizophrenia pathophysiology ( Coyle et al., 2012; Seshadri et al., 2013), and one hypothesis proposes dysruption of cortical interneurons as final common pathway by which several different etiological factors can yield the symptoms characteristic of this disease ( Nakazawa et al., 2012; O’Donnell, 2012). Genetic linkage and association studies have recently begun to identify strong candidate risk genes for schizophrenia, including some ion channels genes ( Askland et al., 2012; Lee et al., 2012; Mulle, 2012; Owen, 2012; Vukadinovic and Rosenzweig, 2012).

#### Calcium channels involvement

Next to BPD, the rs1006737 SNP in *CACNA1C* was found to be a susceptible SNP in schizophrenia in separate studies, though with less statistical power compared to BPD, suggesting possible overlap of genetic risk for both diseases ( Moskvina et al., 2009; Green et al., 2010; Sullivan et al., 2012).

#### Potassium channels involvement

Potassium channels are widely distributed in the CNS and thought to have a role in modulating electrical excitability in neurons, regulating action potential duration and neurotransmitters release. Therefore, it is no surprise that some potassium channel genes have been considered candidate for susceptibility to schizophrenia.

*KCNN3*, encoding the small conductance calcium-activated potassium channel SK3, is located at 1q21, a region reproducibly and strongly implicated in schizophrenia. Several lines of neuroanatomical, physiological, and pharmacological evidence suggested that a SK3 channels may constitute a pharmacological target to improve cognition in schizophrenia and other conditions with cognitive impairment ( Gargus, 2006). In man, SK3 is expressed most abundantly in the ventral tegmental area, in the substantia nigra, the subthalamic nuclei, hippocampus, and amygdala ( Dror et al., 1999). This distribution is consistent with expression in the dopaminergic neurons that contribute to the nigrostriatal and mesolimbic pathways, that are implicated in schizophrenia. Moreover, this brain network abundantly expresses the D_2_ dopamine receptor, the molecular target of typical and atypical antipsychotic drugs. SK channels are the major determinant of the afterhyperpolarization phase (AHP) of the action potential, which regulates the spontaneous frequency and precision of mid-brain dopaminergic neurons. Pharmacological blockade of SK channel enhances bursting activity of these neurons in rats *in vivo* and increases dopamine release. Thus, alterations in the SK3 channels might significantly affect monoaminergic neuron excitability, producing excess dopamine release, a process long implicated in schizophrenia ( Gargus, 2006).

An association between the polymorphic CAG repeat length in the N-terminal coding region of *KCNN3* and schizophrenia has also been questioned, but most results remain quite equivocal. Grube et al. (2011) recently showed that the CAG repeat does not influence susceptibility to schizophrenia but modify its phenotype. Using the Göttingen Research Association for Schizophrenia (GRAS) data collection of patients with schizophrenia, these authors performed a phenotype-based genetic association study of *KCNN3*. They show that long CAG repeats reduce SK3 channel function in HEK 293 cells and, in schizophrenic samples, are specifically associated with enhanced cognitive performance ( Grube et al., 2011). However, interplay of multiple causative factors, perhaps thousands of potential combinations of genes/genetic markers, and an array of different environmental risks, might contribute to the development of a schizophrenic phenotype.

Transgenic mice provided valuable tools to unravel the specific involvement of SK3 channels in synaptic plasticity. Indeed, in the doxycycline-induced conditional SK3-deficient mice some aspects of working/short-term memory are disrupted. In addition, these cognitive deficits in mice are paralleled by reduced brain-derived neurotrophic factor (BDNF) mRNA expression, in the dentate gyrus and CA3 area of the hippocampus, which is also a marker of depression. Hence, the biological substrate for the cognitive impairments in SK3 knock-out mice could conceivably entail reduced trophic support of the hippocampus ( Jacobsen et al., 2009).

The association of *HERG1* gene with schizophrenia stems from the observation that an adverse effect of antipsychotic agents is the acquired long QT (LQT) syndrome, which results from the blockade of the *HERG1* channel. Huffaker et al. (2009) studied two family-based cohorts and three case–control cohorts of European ancestry and reported *HERG1* as a previously undescribed potential susceptibility gene for schizophrenia. The risk alleles predicted increased expression of a brain-selective isoform, KCNH2–3.1 (or Kv11.1–3.1; Huffaker et al., 2009). Atalar et al. (2010) genotyped four SNPs in 7q36.1 region (two SNPs, rs1805123 and rs3800779, located on *HERG1*, and two SNPs, rs885684 and rs956642, at the 3′-downstream intergenic region) and then performed SNP and haplotype association analyses in 84 patients with schizophrenia and 74 healthy controls. Both genotype and allele frequencies of rs3800779 were significantly different between populations. Moreover, two previously undescribed four-marker haplotypes which are located in the chromosome 7 position encompassing *HERG1*, were either over-represented or under-represented in patients compared to controls ( Atalar et al., 2010). Interestingly, the functional characterization of the schizophrenia-associated *HERG1* isoform KCNH2–3.1 revealed altered gating kinetics and lower current accumulation which will result in longer lasting trains of action potentials. Increased expression of these channels in the brain of schizophrenia patients might therefore contribute to disorganize neuronal firing ( Heide et al., 2012). In addition, the genetic variation associated with KCNH2–3.1 expression was reported to influence the therapeutic effects of antipsychotic drugs ( Apud et al., 2012).

An animal model which lacks the KCNH3 channel has been generated. It clarified the critical involvement of this channel in cognitive function. Indeed, *KCNH3* knock-out mice displayed enhanced performance in behavioral tasks related to working memory, spatial reference memory, and attention compared to their wild-type littermates ( Miyake et al., 2009). In addition, these mice had neither the seizures nor the motor dysfunction that are often observed in other potassium channel-deficient animals. Conversely, the overexpression of *KCNH3* in the forebrain caused impaired performance in cognitive tasks. The results indicate that *KCNH3* represent a K^+^ channel that contributes preferentially and bidirectionally to cognitive function ( Miyake et al., 2009). Interestingly, cognitive deficits in schizophrenia have received increasing attention, as they are a core feature of the disease and perhaps the strongest predictor of outcome ( O’Donnell, 2012). Therefore, SK3 and KCNH3 knock-out mice may likely help unraveling the contribution of dysfunction in executive functioning in the etiopathology of schizophrenia.

Yamada et al. (2012) recently identified a SNP (rs3106653) in the *KCNJ3* gene as a result of a GWAS of schizophrenia in the Japanese and Chinese population. *KCNJ3*, also termed GIRK1 or Kir3.1, is a member of the G protein activated inwardly rectifying K^+^ channel group. The analysis of transcript levels in the dorsolateral prefrontal cortex of postmortem brains from patients with schizophrenia and BPD and from healthy controls, revealed significantly lower *KCNJ3* expression in patients compared with controls. These data suggest that this gene is genetically associated with schizophrenia in Asian populations and a more comprehensive analysis extended to other populations will be required ( Yamada et al., 2012).

### AUTISM SPECTRUM DISORDERS

Autism spectrum disorders are characterized by the concomitant occurrence of impaired social interaction, restricted, perseverative and stereotypical behavior, and abnormal communication skills ( Freitag et al., 2010; Devlin and Scherer, 2012). These disorders affect about 1 in 110 individuals, with onset before the age of 3 years ( Devlin and Scherer, 2012). The etiology remains largely unknown, in large part because most cases of ASD arise from a mixture of multiple environmental and genetic influences, making it difficult to forge causal links to behavior ( Freitag et al., 2010; Carter and Scherer, 2013). Moreover, the extreme locus heterogeneity underlying ASD renders the identification of causative or risk associated genes quite difficult, necessitating the analysis of very large cohorts for validation ( O’Roak et al., 2012; Sanders et al., 2012; Carter and Scherer, 2013). Among the genes associated with ASD, most are involved in synaptic plasticity and synaptogenesis, such as neuroligin, neurexin, SHANK, methyl-CpG-binding protein 2 (MeCP2), cyclin-dependent kinase-like 5 (CDKL5), and reelin genes ( Freitag et al., 2010; Sullivan et al., 2012; Carter and Scherer, 2013). Therefore, a developmental deregulation of neuronal networks due to postnatal events, compromising cell differentiation, synaptic formation, and plasticity has been suggested to trigger and/or exacerbate autistic behavior in humans ( Sicca et al., 2011; Bender et al., 2012; Ebert and Greenberg, 2013). In addition, none of these identified genetic variants are associated solely with ASD; they are rather also implicated in intellectual disability, epilepsy, and other psychiatric conditions, suggesting shared biologic pathways ( Carter and Scherer, 2013). This is also the case of some neuronal ion channels implicated in the pathogenesis of autism in association with other clinical features ( Sicca et al., 2011; O’Roak et al., 2012; Sanders et al., 2012).

Here we focus on TS, a form of ASD associated with mutations in a neuronal calcium channel, and Dravet syndrome (DS), which is caused by mutations in neuronal sodium channels and presents a typical epileptic phenotype of infancy; we also report clinical cases with the epilepsy/autism phenotype in which mutations in an inward rectifier potassium channel have been found. Finally, we included psychiatric disorders, caused by antibodies directed against potassium channel complexes.

#### Calcium channels involvement

The *CACNA1C* gene, is the only gene known to be associated with TS, which is therefore considered a monogenic neurodevelopmental disorder ( Paşca et al., 2011). This is a complex disorder that affects multiple organs, characterized by autistic traits and also LQT syndrome, webbed fingers and toes, dysmorphic facial features, and immunodeficiency ( Gargus, 2009). It is a rare childhood disease with less than 20 TS patients identified to date worldwide and an average survival of 2–3 years, the major cause of death being cardiac ventricular fibrillation ( Gargus, 2009; Bidaud and Lory, 2011). In 2004, Splawski et al. (2004) described two mutations, G406R and G402S in the alternative splice exon 8 of Cav1.2 linked to TS. This exon is expressed mainly in brain and heart. The functional characterization of these mutations in heterologous expression systems revealed a gain of channel function, significantly due to loss of voltage-dependent inactivation. This resulted in heightened calcium influx, which may be a contributing factor to the multisystem defects *in vivo* ( Liao and Soong, 2010). Indeed, a sustained entry of calcium into the cardiac myocytes due to impaired inactivation of Cav1.2 channels increases the duration of the action potential, and explains the prolonged QT interval ( Bidaud and Lory, 2011). Interestingly, in brain, this channel mediates a variety of neuronal calcium-dependent processes and regulates calcium influx related to dendritic cells ( Gargus, 2006; Catterall, 2011).

Important results about Cav1.2 channel function and abnormalities came from studies on genetically engineered animal models that showed that Cav1.2 channel plays important roles in remote spatial memories and fear memory extinction ( Bader et al., 2011). The mouse model of TS bears the G406R mutation in the *CACNA1C* gene ( Bader et al., 2011). While homozygous and heterozygous mice are not viable, heterozygous TS2 mice, that are allowed to keep an inverted neomycin cassette, survive through adulthood because the mutation-bearing channel is expressed at lower level. These animals exhibit distinct traits strikingly reminiscent of the entire core triad of ASD: impaired social interaction, impaired vocalization, and restricted and repetitive/perseverative behavior ( Bader et al., 2011). To date, no pre-clinical study has been performed on these animals but it would be of interest to determine whether the various reported behaviors can be modified by L-type channel blockers, either by early intervention or acute treatment.

A fascinating study on patients affected by TS has recently been reported, which supports the hypothesis that ASD arise from defects in connectivity between cortical areas ( Geschwind and Levitt, 2007) and suggests the contribution of increased catecholamine synthesis in the pathophysiology of ASD. Indeed, Paşca et al. (2011) demonstrated that neurons from induced pluripotent stem cells derived from individuals with TS have defects in calcium signaling and activity-dependent gene expression. They also show abnormalities in cell differentiation, including decreased expression of genes that are expressed in lower cortical layers and in callosal projection neurons. In addition, these neurons show abnormal amount of tyrosine hydroxylase and increased production of norepinephrine and dopamine. These findings provide strong evidence that Cav1.2 regulates the differentiation of cortical neurons in humans and that gain of function TS mutations could likely impact neuron development and/or excitability, thus contributing to the presentation of the autistic traits ( Pacşa et al., 2011).

Besides TS, there is no clear evidence to date for a direct link between *CACNA1C* and autism. However, GWAS suggest a possible role of SNPs within calcium channels genes in ASD ( Liao and Soong, 2010). One SNP, rs10848653, is located in *CACNA1C*, two others, rs198538 and rs198545, in *CACN1G*, and a fourth, rs5750860, located in *CACNA1I*, that encode T-type calcium channels ( Strom et al., 2010; Lu et al., 2012).

#### Sodium channels involvement

Various clinical studies assessed the involvement of Nav channels genes in psychiatric developmental diseases including ASD. Early genome-wide scan studies for autism susceptibility genes showed a potential susceptibility region on locus 2q ( Philippe et al., 1999; International Molecular Genetic Study of Autism Consortium [IMGSAC], 2001; Weiss et al., 2003) that includes *SCN1A*, *SCN2A*, and *SCN3A* genes. These results have been confirmed by other laboratories more recently. Indeed, Chen et al. (2010) reported a 2.8-Mb *de novo* cryptic deletion on chromosome 2q24.2/q24.3 detected by array-CGH (comparative genomic hybridization), involving nine genes including *SCN2A* and *SCN3A* in a patient with autistic features, developmental delay, language impairment, mental retardation, and dysmorphic features. These findings suggested that deletion of *SCN2A* and *SCN3A* might be responsible for the autistic features ( Chen et al., 2010). Moreover, a *de novo* missense mutation in *SCN1A* (P1894L), has been identified by using a molecular inversion probe technology in 2,500 individuals, including 1,703 ASD probands and 744 controls. The missense variant is located at a highly conserved position in *SCN1A* and is predicted to be functionally deleterious. The proband is severely affected, with evidence of early onset, possible regression, language delay, epilepsy, and mild intellectual disability ( O’Roak et al., 2011, 2012). In a similar study, Sanders et al. (2012) identified, using whole-exome sequencing of 928 individuals, two probands each carrying two independent non-sense variants disrupting the *SCN2A* gene.

Interestingly, several case reports indicate that the *SCN1A*, *SCN2A*, and *SCN3A* genes are usually associated to epilepsy complicated by neurobehavioral comorbidities, which include cognitive impairment, psychiatric disorders, and social problems. Indeed, Pescucci et al. (2003) reported a 10.4-Mb deletion of 2q24.3eq31.1 spanning a cluster of sodium channel genes including *SCN3A*, *SCN2A*, *SCN1A*, *SCN9A*, and *SCN7A* in a 4-year-old girl with seizures, postnatal growth retardation, microcephaly, facial dysmorphism, developmental delay, stereotypic and repetitive hand movements, and sleep disturbances. Davidsson et al. (2008) described large deletions involving the entire 1.4-Mb sodium channel cluster on chromosome 2q24.3 in patients with seizures, growth and mental retardation, dysmorphic features, and microcephaly. An attempt of genotype–phenotype correlation comes from Bartnik et al. (2011) who proposed that missense mutations of *SCN2A* are responsible for isolated epilepsy, whereas inactivating loss of function mutations or deletion CNVs causing haploinsufficiency of *SCN2A*, can be responsible for a more severe phenotype presenting psychiatric abnormalities, in addition to epilepsy. By using a clinical exon-targeted array-CGH, these authors have identified a *de novo* 112-kb deletion in chromosome 2q24 including portions of the *SCN2A* and *SCN3A* genes in a 25-year-old female with a history of infantile seizure, mental retardation, anxiety disorders, and neurobehavioral and psychiatric abnormalities. The deletion removes exons 1–2 of *SCN2A* and the non-coding exon 1a of *SCN3A*. Although only the non-coding exon 1a of *SCN3A* was deleted in this patient, it cannot be excluded that the function of *SCN3A* is also disrupted because of the deletion of the promoter or transcription binding sites ( Bartnik et al., 2011).

One of the most relevant examples of comorbidity of epilepsy and psychiatric disorders linked to sodium channel dysfunction is DS. DS, also termed severe myoclonic epilepsy in infancy (SMEI), is a childhood epilepsy disorder associated with devastating effects on cognitive and behavioral development, persisting in adulthood ( Bender et al., 2012). While early-life seizures are perhaps the most striking feature of DS, psychomotor, visuospatial, and language development are also impaired, and social development is severely affected, with patients displaying recalcitrance, mood instability, affective indifference, or autistic behaviors. Intellectual disability is reported in the majority of cases ( Bender et al., 2012). Mutations, deletion, and duplication CNVs of *SCN1A*, ranging in size from a single exon to extending beyond *SCN1A*, have been described in patients with DS. Approximately 80% of patients with DS carry loss of function mutations in *SCN1A* ( Catterall et al., 2010; Escayg and Goldin, 2010).

Given that Nav channels are critical for action potential generation and propagation in neurons, it is predictable that mutations in sodium channel might be detrimental to the normal functioning of the nervous system. Whereas it is easier to find a causative association between sodium channels dysfunctions and epileptic attacks, the neural mechanisms responsible for autism spectrum phenotypes, DS and other abnormal neurodevelopmental traits, are less straightforward. In this regard, studies on animal models have helped research in the field by providing important insight into the cellular and neural network mechanisms by which *SCN1A* mutations may cause neural defects ( Yu et al., 2006; Han et al., 2012; Ito et al., 2013).

One major result obtained from *in vitro* studies is the contribution of GABAergic interneurons network to the pathophysiology of DS. Indeed, *SCN1A* has been reported mainly in these GABAergic fast-spiking cells in the neocortex and hippocampus, clustered in the proximal portion of the axon initial segment, precisely a region that is critical for the initiation and generation of action potentials ( Lorincz and Nusser, 2008). Consistently with Nav1.1 expression, it has been shown that loss of function *SCN1A* mutations associated to epileptic disorders cause functional impairments in GABAergic interneurons, reducing GABA release and leading to a loss of appropriate inhibition in related neuronal networks ( Bender et al., 2012). This will create an imbalance between excitation and inhibition in the brain, generating seizures ( Catterall et al., 2010). In addition to epilepsy, impairments in GABAergic interneurons may also likely contribute to the cognitive symptoms observed in patients ( Bender et al., 2012). Actually, these fast-spiking cells are critical for the synchronization and spatiotemporal patterning of neural activity which is essential for the strengthening of cognitive processes ( Fuchs et al., 2007; Murray et al., 2011). These observations have been confirmed in a conditional *Scn1a*^+^^/^^-^ mouse with specific Nav1.1 reduction in GABAergic forebrain interneurons. This mouse represents a clear example of comorbidity of epilepsy and autism. It develops seizure and multiple ASD behavioral phenotypes including hyperactivity, stereotyped behaviors, social interaction deficits, and impaired context-dependent spatial memory. Importantly, these psychiatric symptoms resulted a consequence of impaired GABAergic neurotransmission and not of neuronal damage from recurrent seizures ( Han et al., 2012), as traditionally believed ( Hermann et al., 2008). Indeed, there is a growing body of evidence that other factors are involved in the occurrence of epilepsy comorbidities ( Hermann et al., 2008). In agreement with decreased GABAergic tone, treatment of conditional *Scn1a*^+^^/^^-^ mouse with low-dose clonazepam, a positive allosteric modulator of GABA_A_ receptors, completely rescued the abnormal social behaviors and deficits in fear memory in this mouse model of DS ( Han et al., 2012). These results indicate that low-dose benzodiazepine treatment could be a potential pharmacological intervention for cognitive deficit and autistic symptoms in DS patients.

*In vitro* studies also helped to clarify the cellular and physiological changes in the brain during development, which is of particular relevance to understand the role of *SCN1A* mutations in ASD. Indeed, the developmental origin of several neuropsychiatric disorders is now generally accepted ( Bender et al., 2012; Piper et al., 2012). It is noteworthy that early postnatal development in rodents is highly dependent on changes in Nav brain expression. Nav1.1 expression increases during development reaching a peak during the first 4 weeks of life, while Nav1.2 and Nav1.3 amounts decrease during the second week of life ( Beckh et al., 1989). Consistently, in humans, Nav1.1 expression in the hippocampus and cortex increases during early development, peaking around 7–9 months of age ( Wang et al., 2011). In mice, the Nav1.1 increase parallels both the neurophysiologic growth of fast-spiking interneurons and the development of spatial cognition. Together, the timing of the above developmental changes makes this period especially susceptible to functional deficits affecting neural network activity. Indeed, during this period, mice with complete or partial *Scn1a* deletion show reduced survival, increased propensity to spontaneous or hyperthermia-induced seizures and slowed cognitive development ( Yu et al., 2006; Oakley et al., 2009; Bender et al., 2012; Ito et al., 2013). Moreover, the observation that interneurons of *Scn1a* knock-out mice fail to develop a narrow action potential width characteristic of mature cells, suggests that *Scn1a* expression is important for the conversion from slow to fast-spiking cells ( Yu et al., 2006). Therefore, although the effects of *SCN1A* mutations on brain firing and information processing have not yet been directly tested, a consistent hypothesis is that the deficit in interneurons functioning during development may consistently recapitulate autistic behaviors and cognitive decline in addition to epileptic seizures.

#### Potassium channels involvement

**FIGURE 1:**
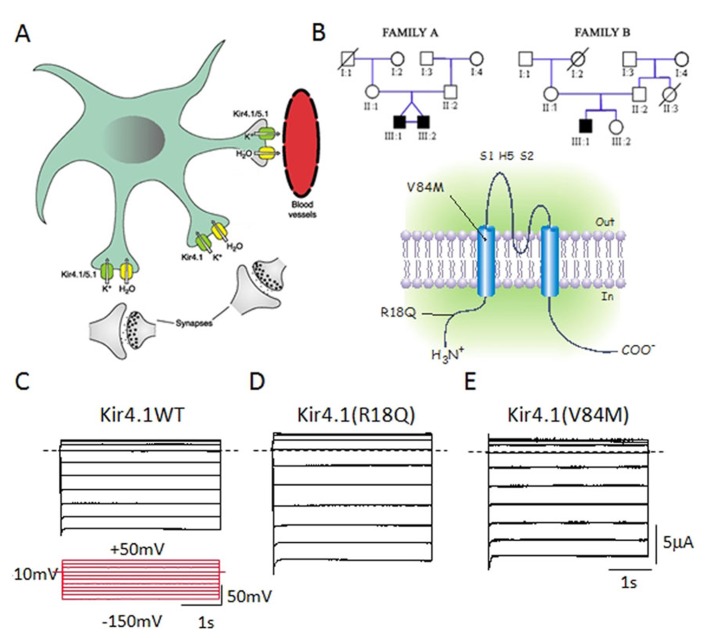
**(A)** Kir4.1 channels localize on brain astrocytes both at perisynaptic and at perivascular processes and control spatial K^+^ buffering. **(B)** *Upper panel*: Pedigrees of two families harboring novel mutations in *KCNJ10* associated to autism/epilepsy phenotype. Squares are males and circles females; solid black symbols represent affected children; slashes denote deceased individual. *Lower panel*: Schematic representation of the human Kir4.1 subunit with the two variants, R18Q and V84M. **(B)** Sample current families recorded from *Xenopus* oocytes expressing equal amounts of Kir4.1 WT **(C)**, R18Q **(D)**, or V84M **(E)** mRNA. Notice that mutant channels show increased current amplitudes compared to wild-type. Horizontal dashed lines indicate 0 current level. The pulse protocol is shown as inset in **(C)**. From Sicca et al. (2011).

There are also case reports of involvement of various types of potassium channels in ASD. A case study reported a decrease in Ca^2^^+^-activated K^+^ channel (BKCa) activity due to a disruption of the BKCa gene (*KCNMA1*) in one subject with ASD ( Laumonnier et al., 2006). A recent linkage analysis study on a large Finnish pedigree proposed *KCNJ10*, coding for the Kir4.1 potassium channel, as a candidate gene for ASD ( Kilpinen et al., 2009). Recently, Sicca et al. (2011) described two heterozygous *KCNJ10* mutations (R18Q and V84M) in three children from two unrelated families with ASD, seizures, and intellectual disability. This study indicated that the molecular mechanism contributing to the disorder relates to an increase in either surface-expression or conductance of the Kir4.1 channel expressed in a heterologous expression system ( Sicca et al., 2011; **Figure 1**). In the brain, Kir4.1 channels are expressed primarily in oligodendrocytes and in astrocytes surrounding synapses and blood vessels, mainly in the cortex, thalamus, hippocampus, and brainstem ( Hibino et al., 2010). Here, these channels control the resting membrane potential of astrocytes and are believed to maintain the extracellular ionic and osmotic environment by promoting K^+^ transport from regions of high [K^+^]_o_, which results from synaptic excitation, to those of low [K^+^]_o_. This polarized transport of K^+^ in astrocytes, referred to as “spatial buffering of K^+^,” is essential for normal neuronal activity, excitability, and synaptic functions ( Chever et al., 2010). Dysfunction in the astrocytic-dependent K^+^ buffering has been suggested as a common mechanism contributing to seizures as well as to ASD behavioral traits, by altering neuronal excitability and synaptic function, and may represent a new target for therapeutic approaches ( Sicca et al., 2011). Fifty to seventy percent of autistic children show some degree of intellectual disability ( Matson and Shoemaker, 2009). Both cognitive and autistic features could be tied to postnatal developmental brain injury ( Hong et al., 2005) and Kir4.1 channel activity shows a profound developmental regulation, which correlates with both cell differentiation and the developmental regulation of extracellular K^+^ dynamics ( MacFarlane and Sontheimer, 2000; Neusch et al., 2001). Therefore, early abnormal functioning of mutant Kir4.1 channels may impact the correct brain development, contributing to intellectual and psychiatric signs. Moreover, astrocyte-released neuroactive substances govern several functions including neuronal excitability, excitatory and inhibitory synaptic transmission, and plasticity ( Yang et al., 2003; Zhang et al., 2003; Pascual et al., 2005) as well as synaptogenesis and neuronal wiring ( Collazos-Castro and Nieto-Sampedro, 2001; Ullian et al., 2004) which will be compromised by Kir4.1 mutations ( Sicca et al., 2011). Therefore, even though a direct causative link has still to be ascertained, different hypotheses related to potassium homeostasis, cell differentiation, and synaptic plasticity have been evoked to correlate Kir4.1 mutations to epilepsy and ASD. Several lines of evidence from animal models of ASD come in support of the clinical studies. Interestingly, an up-regulation of Kir4.1 has been found in locus coeruleus neurons of an animal model of Rett syndrome, the MECP2-null mice ( Zhang et al., 2010b). Here, Kir4.1 overexpression might impair noradrenergic modulation, leading to the autistic behaviors seen in Rett syndrome ( Zhang et al., 2010b).

Interestingly, another disorder with molecular and clinical features different from the above described cases, has been associated to Kir4.1 mutations. Indeed, independent clinical studies unraveled the involvement of Kir4.1 in EAST/SeSAME syndrome, that presents with a unique set of symptoms including epilepsy, ataxia, mental retardation, hearing loss, and electrolyte imbalance related to renal salt loss ( Bockenhauer et al., 2009; Scholl et al., 2009; Williams et al., 2010). Genetic screening revealed that EAST/SeSAME patients are homozygous or compound heterozygous for novel missense mutations in *KCNJ10* or present a deletion of the C-terminal half of the protein Kir4.1 ( Williams et al., 2010). These studies showed that each of these mutations compromises the function of both homomeric Kir4.1 and heteromeric Kir4.1/Kir5.1 channels and that Kir4.1 function is always reduced by distinct mechanisms ( Sala-Rabanal et al., 2010). Furthermore, almost all mutations do not largely impair the ability of these channels to be expressed at the plasma membrane indicating the potential utility of Kir channel openers for SeSAME syndrome therapy ( Tang et al., 2010). The localization and physiological role of Kir4.1 channels in renal tubules and in the inner hear ( Hibino et al., 2010) well explain why loss of channel function causes sensorineural deafness and the electrolyte imbalance reported for EAST/SeSAME patients. Less trivial would be to ascertain the link between Kir4.1 reduction and both seizure susceptibility and cognitive dysfunctions. These clinical features, as discussed before, might be at least in part attributed to channel expression and function in neurons and astrocytes during development. Relevant clues can be drawn from Kir4.1 knock-out mice, which display seizure, hearing loss, altered K^+^ transport in hippocampus, impaired glutamate uptake by astrocytes, motor impairment due to hypomyelination, but not clear signs of cognitive decline as in affected patients bearing Kir4.1 mutations ( Djukic et al., 2007).

Other potassium channels have been implicated in developmental disorders. Birk Barel mental retardation dysmorphism syndrome is a maternally transmitted genomic-imprinting syndrome of mental retardation, hypotonia, and unique dysmorphism with elongated face ( Barel et al., 2008). The disease was mapped on chromosome 8q24 and was caused by a missense mutation (G236R) in the maternal copy of *KCNK9* which encodes K_2P_9.1, a member of the two pore-domain potassium channel (K_2P_) subfamily ( Kim et al., 2000). The mutant subunits gave rise to non-functional homodimeric channels and showed a dominant-negative effect when co-expressed with wild-type or with K_2P_3.1 ( Barel et al., 2008). K2P potassium channels carry leak or background currents that are mostly time- and voltage independent. These leak currents shape the duration, frequency, and amplitude of action potentials, regulate cell excitability by modulating membrane resting potential, and are mainly expressed in the cerebellum ( Kim et al., 2000; Goldstein et al., 2001; Kang et al., 2004). Due to its localization and function it is reasonable that the K_2P_9.1 loss of function during brain development might specifically disrupt the efficiency of the cerebro-cerebellar pathways, resulting in cognitive deficits and deterioration in muscle strength and function ( Barel et al., 2008).

#### Autoimmune psychiatric disorders

A group of neuropsychiatric disorders affecting both adults and children is caused by auto antibodies targeting macromolecular complexes containing a potassium channel ( Irani and Vincent, 2011; Kayser and Dalmau, 2011). The antibodies are directed against the Kv1 voltage-gated potassium channels (VGKC) and associated proteins including leucine-rich glioma-inactivated 1 protein (LGi1) and contactin-associated protein 2 (Caspr2). Therefore, they are better termed “VGKC complex” antibodies ( Kayser and Dalmau, 2011; Lin et al., 2012). The psychiatric symptoms present in patients with VGKC autoimmunity include panic attacks, obsessive–compulsive behaviors, and cognitive alterations ( Irani and Vincent, 2011; Somers et al., 2011).

Limbic encephalitis, a prototypic autoimmune neuropsychiatric disorder, is recognized by neurologists and psychiatrists by its subacute onset and rapid progression of cognitive, mood, and behavioral symptoms ( Kitten et al., 2011; Somers et al., 2011). Classically, symptoms evolve over days to weeks and include psychiatric manifestations as diverse as irritability, depression, hallucinations, and personality disturbances, with neurocognitive changes in the form of short-term memory loss, sleep disturbances, and seizures ( Kayser and Dalmau, 2011). Somers et al. (2011) described the neuropsychiatric spectrum of Kv channel complex autoimmunity among 67 seropositive patients and found a significant correlation between severe neuropsychiatric presentations (such as confusion, memory impairment, personality change, depression, anxiety, visual hallucinations, delusions, and sleep disorders) and higher autoantibody values. Of 15 who received immunotherapy, 67% improved. Improvements were most evident in patients treated early, which emphasizes the need for early diagnosis and immunotherapy initiation ( Somers et al., 2011).

Some cases of fever-induced refractory epileptic encephalopathy in school-age children (FIRES) may have an immunological basis ( Haberlandt et al., 2011; Suleiman et al., 2011). This is a clinically recognized epileptic encephalopathy of unknown etiology in which onset of epilepsy is accompanied by a dramatic cognitive decline, behavioral difficulties, and, in some cases, the evolution of neurological signs. Illingworth et al. (2011) described a case of FIRES in a 4-year-old boy with significant attention, memory, and word-finding difficulties, which was associated with elevated Kv channel complex antibodies and a significant clinical and immunological response to immunomodulation.

Studies from genetically modified mouse models ( Smart et al., 1998) and from disease causing mutations in heterologous expression systems ( D’Adamo et al., 1999; Imbrici et al., 2011) contributed to unravel the functional role of Kv1 channels in the brain and the consequences of an altered activity on the brain areas where they are expressed. Loss of function mutations in Kv1.1 channels cause attacks of motor incoordination due to altered GABAergic neurotransmission at the cerebellar basket cell–Purkinje cell synapse ( D’Adamo et al., 1999). Some individuals bearing Kv1.1 mutations also experience epilepsy and cognitive decline which may result from altered glutamate neurotransmission at the hippocampal CA3 region, as a consequence of impaired Kv1.4–1.1/Kvβ1.1 channel inactivation ( Geiger and Jonas, 2000; Imbrici et al., 2006). Consistently, purified immunoglobulin G from one individual with limbic encephalitis reduced VGKC function at mossy fiber-CA3 pyramidal cell synapses and increased cell excitability. Moreover, α-dendrotoxin, a selective Kv1.1, 1.2, and 1.6 subunit antagonist of VGKC, mimicked the limbic encephalitis immunoglobulin G-mediated effects ( Lalic et al., 2011). These studies support the role of Kv1.1 channels in brain disorders and the contribution of hippocampus impairment to the genesis of epilepsy and cognitive defects.

Among the autoimmune neuropsychiatric disorders involving potassium channels, the Pediatric Autoimmune Neuropsychiatric Disorders Associated with Streptococcal Infection (PANDAS) presents neuromuscular symptoms, relapsing–remitting tics, and obsessive–compulsive signs, together with group A β-hemolytic streptococcal pharyngotonsillitis ( Snider and Swedo, 2003; Squintani et al., 2010). An abnormal production of auto antibodies against PK has been recognized among the pathogenic factors ( Kansy et al., 2006). In order to clarify the mechanism of the disease, Mele et al. (2012) proposed that KATP/PK complex might represent a novel pathogenic target in PANDAS. Indeed, patch clamp experiments in neuronal cell lines and muscle fibers revealed that KATP channels are functionally associated to the PK and that the ATP produced by the PK likely inhibits KATP channel conductance ( Mele et al., 2012). Moreover, anti-PK antibodies have the potential to alter membrane-associated glycolytic ATP production and display a dual mode of action on neuronal KATP channels, potentiating KATP currents in the short-term and reducing KATP currents in the long-term incubation. This promotes cell survival and neuronal death, respectively. These results suggest that in affected patients KATP channel opening or closing by anti-PK antibodies, may change the resting membrane potential, the spike generation during action potentials and alter the neuronal overall excitability. Consequently, the extracellular levels of several neurotransmitters, including dopamine, serotonin, glutamate, and GABA will likely be affected ( Milton and Lutz, 2005; Chan et al., 2007; Soundarapandian et al., 2007). Such changes might be relevant in the cortico-striato-thalamo-cortical circuits, which modulate movement, executive functions, and emotional control and might contribute to the pathophysiology of the disease (tics and obsessive–compulsive symptoms; Squintani et al., 2010).

## ION CHANNELS AS PHARMACOLOGICAL TARGETS IN NEUROPSYCHIATRIC DISORDERS

Given the pivotal role played by ion channels in psychiatric disorders ( Gargus, 2006, 2009; Liao and Soong, 2010; Sicca et al., 2011; Askland et al., 2012; Mele et al., 2012; O’Roak et al., 2012) and their specific function and localization in brain areas implicated in mental illness ( Hibino et al., 2010; Catterall, 2012; Jan and Jan, 2012), many channel subtypes have emerged as critical targets for therapeutics. Moreover, commercially available medications for psychiatric diseases result to bind neuronal ion channels, thus suggesting that these proteins may be involved in the therapeutic action of conventional drugs ( Ohno et al., 2007; Eijkelkamp et al., 2012; Perucca and Mula, 2013). Indeed, it is noteworthy that activation or inhibition of different ion channels can alter the extracellular levels of several neurotransmitters, including dopamine, serotonin, glutamate, and GABA, which are notoriously implicated in psychiatric disorders ( Aldana and Sitges, 2012; Mele et al., 2012). In addition, medications used to treat mental disorders work differently for different people, are marginally effective and present several side effects. Examples are first- and second-generation antipsychotic drugs that improve positive symptoms, but are poorly active for negative symptoms and not at all effective for cognitive deficits ( O’Donnell, 2012). Therefore, for the mentioned considerations, novel molecules acting as selective ion channels blockers and openers are under development. Drug design and development are being greatly assisted by the recently identified crystal structures of several ion channels, from high-throughput screening methods and, finally, from transgenic mice for ion channel genes and pharmacologically induced animal models of psychiatric disorders. Here we summarize the main pharmacological strategies directed to block or activate calcium, sodium, and potassium channels.

### CALCIUM CHANNELS PHARMACOLOGY

These channels have become drug targets for a range of cardiovascular and neurological diseases. The clinical value of calcium channel inhibitors in mental illness has been considered following experimental results showing an increased expression of the *CACN1AC* transcript and/or an increase in calcium channel activity in some psychiatric conditions. Moreover, inhibition of Cav channels probably translates into a reduction in excitatory neurotransmission, which may be ultimately responsible for positive effects on mood and behavior. Despite their potential, calcium channel blockers have been studied clinically, in psychiatric disorders such as mood disorders and substance abuse/dependence, yielding conflicting results. There have been small clinical trials in the past suggesting benefit of verapamil and other calcium channel inhibitors for some patients affected by BPDs, but the data have been inconsistent and limited. Interestingly, nicardipine, a dihydropyridine calcium channel blocker, was found to enhance the antidepressant action of electroconvulsive therapy in 26 patients affected by major depression ( Dubovsky et al., 2001). Nevertheless, to date, there is not definitive evidence of efficacy of any typical calcium channel antagonists for neuropsychiatric disorders ( Casamassima et al., 2010). Other strategies to reduce calcium currents have been explored. Recently, Paşca et al. (2011) reported the beneficial effect of a treatment with roscovitine, a cyclin-dependent kinase inhibitor and atypical L-type channel blocker, on neurons from induced pluripotent stem cells derived from individuals with TS, resulting from gain of function Cav1.2 mutations. Besides targeting the Cavα_1_ subunit, recent calcium channel modulators are directed toward the accessory α_2_δ subunit. As a recent study showed, the α_2_δ ligand pregabalin administration prevented the appearance of depression-like behaviors induced by chronic restraint stress, and promoted hippocampal neurogenesis in adult stressed mice. The α_2_δ_1_ subunit and the nuclear factor-κB signaling pathway have been suggested to play a role in drug-mediated proneurogenic effects. These pharmacological activities of α_2_δ ligands may help to explain their therapeutic activity as supplemental therapy for major depression and depressive symptoms in post-traumatic stress disorder and generalized anxiety disorders ( Valente et al., 2012).

### POTASSIUM CHANNELS PHARMACOLOGY

Researchers at Wyeth disclosed compounds that disrupt interaction between Kv1α and β subunits and thus prevent Kv1.1 N-type inactivation. Kv1 channels in brain contribute to hyperpolarize the neuron following depolarization. In the hippocampus Kv1.1 is co-expressed with Kvβ1 (and other β subunits), which converts the non-inactivating Kv1.1 into a transient, fast inactivating current, reducing its ability to hyperpolarize the cell and thus increasing glutamate release and neuronal excitability. Despite the current development status of this therapeutic strategy is unknown, these molecules may be useful for reducing neuronal hyperexcitability in diseases such as epilepsy and neuropathic pain but could also be adjuvant in diseases presenting with cognitive symptoms of hippocampal origin ( Lu et al., 2008).

Promising therapeutic interventions target SK channels due to their wide distribution in the CNS and contribution to neuronal excitability. SK channels activation has been proposed in several disorders involving loss of synaptic plasticity, including loss of memory and learning disabilities, where physiological neurotransmission needs to be restored ( Gargus, 2006). Modulation of SK channels has been suggested as a novel protective strategy in neurological disorders where neuronal cell death and neuroinflammatory processes are prominent, included schizophrenia ( Dolga and Culmsee, 2012). Genetic and physiological data support the role of SK channels in the neuropathology of this disease. Among SK channels, the SK3 isoform, controls pacemaker frequency and precision in dopaminergic neurons and seem to play a role in dopamine release in mid-brain pathways. Therefore, this physiological function of SK channels could be explored and SK openers proposed as a novel pharmacological strategy in addition to classic antipsychotic drugs. Interestingly, the role of SK channels in controlling the pacemaking activity of neurons has been exploited in episodic ataxia type 2, a neurological disease caused by loss of function mutations in P/Q type calcium channels. Non-selective SK channels activation with 1-EBIO or chlorzoxazone repaired the precision of Purkinje cell pacemaking and ameliorated the cerebellar signs in a mouse model of episodic ataxia. Indeed, a reduced activation of SK channels, due to decreased calcium entry into Purkinje cells, has been shown to contribute to the disease ( Walter et al., 2006; Alviña and Khodakhah, 2010).

KCNQ/Kv7 potassium channels, the molecular counterpart of brain M-current, have also received considerable interest by pharmaceutical market over the past decade. These channels limit repetitive firing and cause spike-frequency adaptation ( Rogawski, 2000), modulate synaptic plasticity and are inhibited by ACh through the muscarinic receptors. Early studies with M-current inhibitors like linopirdine demonstrated improvements in learning and memory performance in animals ( Fontana et al., 1994). A recent study with XE991, a second-generation M-current blocker, in healthy mice has shown that the improvement in cognitive abilities may be achieved by altering basal hippocampal synaptic activity and by decreasing the levels of KCNQ/Kv7.3 protein, a pivotal subunit for the M-current. Furthermore, XE991 can revert both the cognitive impairment associated with acetylcholine depletion, and the neurodegeneration induced by kainic acid ( Fontan-Lozano et al., 2011). Consequently, inhibition of the hippocampal M-current has been proposed as a general strategy to enhance cognitive performance in healthy and aging individuals, as well as in those with neurodegenerative diseases ( Brioni et al., 1993; Jentsch, 2000). However, clinical efficacy studies investigating improvement of cognitive function have not yet been reported.

At the same time, a number of pharmaceutical companies are involved in the development of KCNQ channel activators to treat neurological diseases. The first agent proven to enhance M-current activity was retigabine. When examined *in vivo*, retigabine exhibited anticonvulsant activity in a broad range of seizure models and was successful in a number of clinical trials in humans. After retigabine, others Kv7.2/Kv7.3 activators such as ICA-27243 and ICA-105665 have been developed as anticonvulsant. Several findings support the use of these compounds also for the treatment of neuropsychiatric disorders. Kv7 channel activation strongly suppresses dopaminergic activity and it is well known that altered function of the dopaminergic system is associated with neuropsychiatric disorders ( Hansen et al., 2008; Cousins et al., 2009; Gonul et al., 2009). Moreover, both selective and non-selective Kv7.2/7.3 activators exhibit efficacy in animal models of anxiety, mania, BPD, ADHD, and schizophrenia ( Hansen et al., 2008). Indeed, pharmacological stimulation of heteromeric Kv7.2/Kv7.3 channels showed promising results in an amphetamine and chlordiazepoxide induced hyperactivity model of mania ( Redrobe and Nielsen, 2009). In addition, neuroimaging studies in bipolar patients with mania revealed alterations in metabolic activity in corticolimbic areas and demonstrated that Kv7 channel activation with retigabine and ICA-27243 reduces baseline cerebral glucose metabolic activity ( Kristensen et al., 2012). These two Kv7 channel openers dose-dependently increased GSK3β in the prefrontal cortex and hippocampus, regions implicated in the emotional and cognitive aspects of mental illness. The association of GSK3β with Kv7 channel function is relevant, as GWAS have identified polymorphisms implicating GSK3β signaling cascades in BPD ( Baum et al., 2008). Moreover, researchers at Lundbeck demonstrated, in a conditioned avoidance response paradigm model of antipsychotic activity, that retigabine could inhibit avoidance responses, an effect blocked by the Kv7 inhibitor XE-991184. Retigabine was also able to inhibit hyper-locomotor responses in phencyclidine-sensitized animals, which is often considered as a disease model for schizophrenia ( Scotty et al., 2009). Interestingly, retigabine has recently been approved for adjunctive therapy in partial-onset seizures ( French et al., 2011), and because bipolar patients often experience therapeutic benefit from treatment with anticonvulsant drugs ( Altamura et al., 2011), this also implies that Kv7 channel openers may also prove to have clinical utility in the treatment of BPDs. Therefore, these experimental and pre-clinical data support the hypothesis that positive modulation of Kv7 channel function restores several key signaling pathways in psychosis similarly to standard drugs, like lithium and valproate, and emphasizes the potential benefit of Kv7 channel openers in the treatment of these diseases.

Kv11.1 (HERG) plays a crucial role in cardiac repolarization, especially in the later phases of the action potential due to its unique kinetics. As it is a promiscuous channel and binds to a structurally diverse set of small molecules, many drugs have been removed from the market or terminated during clinical development because of cardiac side effects. It is now common practice to assess the HERG blocking liability of compounds before they are taken to the clinic. In the last few years several HERG activators were developed and were considered potential therapeutics for antiarrhythmia ( Wulff et al., 2009). As example, the small compound NS1643 has been shown to activate native and heterologously expressed Kv11.1, Kv11.2, and Kv11.3 channels ( Bilet and Bauer, 2012; Bagal et al., 2013). Besides its relevance in cardiac physiology, relative overexpression of a primate-specific, brain isoform of Kv11.1 (*KCNH2–3.1*), which lacks an N-terminal domain crucial for slow deactivation and therefore induces high-frequency, non-adaptive firing patterns in cultured cortical neurons, has recently been linked to an increased risk of schizophrenia ( Huffaker et al., 2009). The authors of this study speculate that isoform-specific inhibitors might be useful for the treatment of schizophrenia. Alternatively, selective Kv11.1 channel activators could compensate for the reduced expression of the full-length isoform. The physiological role of the different Kv11 channels in the CNS is still under investigation and at present, it is unclear whether Kv11 channels are suited as pharmacological target to influence neuronal excitability *in vivo*. Nevertheless, drugs specifically affecting a given Kv11 subunit would probably diminish the unwanted side effects either in the heart or in the brain.

Kir4.1 channel plays a critical role in K^+^ homeostasis in the human CNS and mutations in this channel type gives rise to adverse phenotypes with loss and gain of function. No selective Kir4 modulators have thus far been disclosed. However, investigators studying antidepressant drug modulation of glial cell function have demonstrated that many of these drugs inhibit Kir4 with relatively low affinity. Indeed, selective serotonin reuptake inhibitors (SSRIs), fluoxetine and escitalopram, and tricyclic antidepressant, nortriptyline, are known to block inward rectifier potassium channels. Interestingly, Ohno et al. (2007) found that SSRIs preferentially inhibit Kir4.1 channel rather than Kir1.1 and Kir2.1. The common structure–activity relationship between channel families suggests a common Kir channel antidepressant binding site. Two amino acids, Thr128 and Glu158, on transmembrane domain 2 of Kir4.1 are critical for the drug inhibition of the current by fluoxetine and nortriptyline ( Furutani et al., 2009). Intriguingly, some serotonin reuptake inhibitors, are used for the clinical management of repetitive and challenging behaviors in children with autism ( Blenner et al., 2011) and an up-regulation of Kir4.1 channels has been recently associated to the presentation of autistic traits in children ( Sicca et al., 2011). This may suggest that astroglial Kir currents might be involved in their pharmacological action. At the same time, it is also formally possible that Kir4 channel activators may promote glial cell K^+^ uptake and thereby exhibit some antiepileptic activity required in SESAME/EAST syndrome.

Also GIRK channels are inhibited by tricyclic antidepressants and this effect may contribute to some of the therapeutic effects and to the adverse effects, especially seizures and atrial arrhythmias in overdose, observed in clinical practice ( Kobayashi et al., 2004). Interestingly, neuronal GIRK channels are indirect target of antipsychotic drugs, being one of the main effectors of serotonergic and glutamatergic neuroreceptors activation ( Fribourg et al., 2011).

The drug discovery of novel antipsychotic drugs, has recently also demonstrated the role of neuronal and astrocytic KATP channels as interesting therapeutic target, in addition to D2 and 5-HT2A neuroreceptors. Indeed, these channels are expressed in the neural circuits that are implicated in the pathophysiology of schizophrenia and are modulated by dopamine receptors ( Lin et al., 1993). Moreover, diazoxide, an ATP-sensitive potassium channel opener, has been tried in the clinic as an adjunctive treatment with haloperidol. It potentiated the effects of haloperidol on the positive and general psychopathological symptoms of schizophrenia. A novel brain KATP channel opener, iptakalim, has been proposed by Sun et al. (2010) as a novel antipsychotic molecule. They showed that in association with clozapine in rats, iptakalim reduced amphetamine- and phencyclidine-induced hyperlocomotion, disrupted selectively conditioned avoidance responding and increased c-Fos expression in the medial prefrontal cortex, nucleus accumbens, and lateral septal nucleus ( Sun et al., 2010). This supports a role of KATP in the treatment of schizophrenia.

Activators of K2P channels are anticipated to have a therapeutic potential to treat neurological and cardiac diseases. Currently, there are relatively few compound classes that have been shown to selectively modulate the different subsets of this class of potassium channels ( Bagal et al., 2013).

### SODIUM CHANNELS PHARMACOLOGY

A variety of approved drugs are modulators of neuronal sodium channels for the treatment of clinical conditions associated with abnormal cell excitability, mainly neuropathic pain and epilepsy ( Mantegazza et al., 2010). Some non-selective sodium channel blockers, besides acting as antiepileptic drugs, can also be mood stabilizers and antidepressants. Lamotrigine, a known sodium channel blocker, has been shown to prevent with great efficacy episodes of depression, through a positive effect on corticolimbic network which is disrupted in BPD. In healthy volunteers, this drug has shown a specific facilitatory effect on the prefrontal cortex while, *in vitro*, it enhanced the power of gamma frequency network oscillations induced by kainic acid in the rat hippocampus ( Large et al., 2009). Lamotrigine has indirect antiglutamatergic effects by acting at Nav channels to stabilize neuronal membranes and glutamate release. Interestingly, this drug is also effective for the treatment of Lennox–Gastaut syndrome, a rare and intractable form of childhood epilepsy associated with learning difficulties. Phenytoin is an established antiepileptic drug with a robust capacity to bind to and prolong the inactivation of mammalian, Nav channels. Small randomized studies suggest that phenytoin may be useful in the treatment of BPD (acute therapy for manic episodes and maintenance treatment), major depressive disorder, and impulsive aggression ( Perucca and Mula, 2013). A prototypical sodium channel-blocking antiepileptic drug, carbamazepine is approved by the Food and Drug Administration for the treatment of acute manic and mixed episodes of BPD. This drug appears to be effective for maintenance treatment of BPD, although to a lesser extent compared with lithium; it may also be effective in unipolar depression, whereas its utility in schizophrenia is still uncertain ( Perucca and Mula, 2013). These effects, however, cannot be explained solely on the basis of the known sodium channel block of each anticonvulsant, and other mechanisms or targets are likely to be implicated. Indeed, lamotrigine also acts on other molecular targets, such as the hyperpolarization-gated cationic current in dendrites of pyramidal neurons, N- and P-type Cav channels in cortical neurons and neocortical potassium currents ( Eijkelkamp et al., 2012).

Interestingly, typical antidepressant drugs have gained attention for their ability to block sodium channel. Importantly, among the pathophysiological mechanisms of mood disorders, evidence that sodium homeostasis is altered also exists. Early studies found that erythrocyte and whole body intracellular sodium concentrations are increased in patients with depression and BPD. Furthermore, several effective mood-stabilizing and antidepressant treatments reduce intracellular sodium concentrations or inhibit, through blockade of Nav channels, sodium influx ( El-Mallakh and Huff, 2001). In a mouse model of seizure, the SSRI fluoxetine was shown to behave as an anticonvulsant in a dose- and time-dependent manner, and this action was accompanied by strong inhibition of persistent sodium current and impairment of repetitive firing. These findings suggest that the effect of fluoxetine on active membrane properties is similar to that of many antiepileptic drugs, and this action may contribute to anticonvulsant effects ( Igelström and Heyward, 2012). In an elegant study, Aldana and Sitges (2012) demonstrate that besides the inhibition of 5-HT reuptake, sertraline is an effective inhibitor of pre-synaptic sodium channels controlling neurotransmitter release. Of note, blockade of Nav channels also results in inhibition of glutamate release ( Farber et al., 2002), which is itself associated with positive effects on mood and behavior.

So far sodium channel openers have been considered only as toxic compounds. However, a selective opener could have clinical applications in some disorders. For example, a Nav channel opener selective for Nav1.1, whose loss of function can cause DS and other genetic epileptic syndromes, might be a particularly effective antiepileptic drug for these syndromes. Similarly, compounds able to selectively increase Nav1.1 expression levels would probably be even more efficacious in these cases, but little has been done to develop drugs with this mechanism of action ( Bagal et al., 2013).

## CONCLUSION

Voltage-gated ion channels, as the main determinants of intrinsic neuronal excitability, are implicated in many inherited and acquired diseases and thus, they are particularly appealing targets for pharmacological intervention. The reported research studies suggest that ion channels genetic variants are potential mechanisms of risk or causative factors for BPD, schizophrenia, and ASDs ( Gargus, 2009; Liao and Soong, 2010; Mele et al., 2012; Somers et al., 2011; Askland et al., 2012; O’Roak et al., 2012). The wide distribution of ion channels in brain and specifically, their localization in hippocampus, prefrontal cortex, and amygdala, brain areas which are disrupted in major psychiatric disorders, further support the association between these proteins and mental illness ( Gargus, 2006; Aldana and Sitges, 2012; Bender et al., 2012). Indeed, the calcium channel Cav1.2 has been strongly associated to the susceptibility to BPD and schizophrenia ( Liao and Soong, 2010), and several laboratories report a link between schizophrenia and SK3 channels ( Gargus, 2006). Mutations in Cav1.2 are the sole cause of TS ( Bidaud and Lory, 2011) and genetic variants in brain sodium channels are responsible of several childhood neuropsychiatric disturbances ( Bender et al., 2012; Sanders et al., 2012). Kir4.1 is involved in developmental syndromes with epilepsy, mental retardations, deafness, and renal dysfunctions ( Sala-Rabanal et al., 2010; Sicca et al., 2011). Antibodies against Kv1.1 channels have been found in patients with psychiatric symptoms ( Illingworth et al., 2011; Somers et al., 2011).

As commonly observed for other genetic diseases, neuropsychiatric disorders and ASD in particular, present extreme genetic heterogeneity, involving hundreds of genes, necessitating the analysis of very large cohorts of patients and robust statistical methodologies for validating results ( Sanders et al., 2012). The scenario is further complicated by the fact that *de novo* mutations, may also contribute substantially to the genetic etiology of ASD and schizophrenia ( Mulle, 2012; O’Roak et al., 2012). Together with the poor number of patients sometimes available for studies, these factors make the genetic and clinical diagnosis often quite difficult.

As clear from the reports here summarized, in the majority of clinical cases, ion channel variants are generally identified in individuals expressing neurologic or other syndromic features as well as psychiatric features (e.g., epilepsy and psychiatric symptoms). Epilepsy is very common among clinical findings, usually associated to autistic traits, major psychosis, and intellectual disability. The overlapping etiopathology between epilepsy and psychiatric illness has been discussed in the text for DS, where epilepsy occurs together with autistic behaviors, but can be likely extended to similar diseases, such as those caused by other ion and neurotransmitter operated channels ( Derry et al., 2008; Liao et al., 2011; Sicca et al., 2011). Relevant information about the etiopathology of DS comes from studies on animal models which selectively lack the Nav1.1 gene in cortical GABAergic interneurons ( Han et al., 2012). Animal studies have revealed that loss of Nav1.1 function causes impaired firing of GABAergic interneurons relative to pyramidal cells. Actually, the most supported hypothesis is that dysfunction of GABAergic interneurons may account both for recurrent seizures and for cognitive defects/autistic behaviors. Indeed, GABAergic neurons serve a complex role in normal brain functions: beyond balancing excitation with inhibition, they are pivotal for the temporal coordination of neural activity subserving cognitive functions ( Fuchs et al., 2007; Bender et al., 2012). Therefore, the cognitive impairment is likely not only attributable to brain injury due to severe and recurrent seizure, but also to an anomalous functioning of cortical interneurons. Examples may include alterations in theta and gamma oscillations, and loss of coordination of pyramidal cell ensembles involved in information processing ( Bender et al., 2012). A new literature is now under way, linking cognitive abnormalities in different diseases directly to indices of structural, functional, metabolic, and other neurobiological markers of cerebral integrity, independent of their association with clinical epilepsy features ( Hermann et al., 2008). In addition and no less important, the biological maturity of interneurons parallels Nav1.1 expression increase during development, indicating the importance of this (and others) ion channel for setting brain networks during growth. This can be also the case for Kir4.1 mutations in children affected by epilepsy and ASDs. An abnormal functioning of the astrocyte-neuron synapse during development can account for the overlapping phenotype observed in these patients ( Sicca et al., 2011). As well, alteration of cortical interneurons activity during development supports one current pathophysiological view of schizophrenia ( O’Donnell, 2012). This finding holds up the assumption that the disruption of a specific brain circuitry due to ion channels defects (but also due to other causes) during development may account for the psychiatric disease susceptibility or progression. Indeed, dysregulation of activity-dependent signaling pathways controlling synaptic function and allowing neuronal circuits to respond dynamically to experience, may have a key role in the etiology of ASD ( Ebert and Greenberg, 2013).

Further studies are necessary to fully characterize the exact composition of ion channels complexes as well as their specific cellular localization and function in the brain. Of particular interest are transgenic technologies that allowed the engineering of mouse models mimicking different kinds of monogenic heritable forms of psychiatric disorders. These transgenic models provide excellent opportunities to explore in detail the cellular and molecular mechanisms underlying mental illness pathology and to test novel therapeutic intervention.

Indeed, the involvement of ion channels in mental illness represents an attractive alternative target for the pharmaceutical drug discovery, committed to overcome the limits of common neuropsychiatric drugs such as the adverse effects and limited responsiveness. The recent advent of high-throughput electrophysiology, as well as the emerging structural information of ion channel proteins, helps pharmaceutical research at identifying selective ion channel modulators with therapeutic potential in neuropsychiatric syndromes. As discussed in this review, the pharmacological profile of classic calcium channel blockers in psychiatric disorders has still to be ascertained ( Casamassima et al., 2010); instead calcium channel modulators targeting the α_2_δ subunits are showing positive results ( Valente et al., 2012). Among potassium channels ligands, particular interest has been devoted to SK channel openers with potential antipsychotic properties. These channels are indeed involved in the control of fire frequency of dopaminergic neurons, one of the principal neuronal networks disrupted in schizophrenia ( Gargus, 2006). Inhibition of the hippocampal M-current has been proposed as a general strategy to improve cognitive performance ( Fontan-Lozano et al., 2011); on the other hand M-current openers in prefrontal cortex and hippocampus resulted helpful in mouse model of BPD and schizophrenia. These pre-clinical data indicate that M-current activation restores several key signaling pathways in psychosis similarly to standard drugs, like lithium and valproate ( Kristensen et al., 2012). Kir channels are molecular targets of several antidepressants, thus suggesting that these channels may contribute to the therapeutic action (or adverse effect) of these drugs ( Ohno et al., 2007; Furutani et al., 2009). In addition, KATP potassium channels openers have been tested as adjuvant in combination with haloperidol and clozapine in the treatment of schizophrenia ( Sun et al., 2010). Sodium channels blockers such as typical anticonvulsants display antidepressant activity ( Perucca and Mula, 2013), while common antidepressants have been shown to block sodium channels, thus proposing the role of Nav channels in the etiology of depression ( Aldana and Sitges, 2012).

Research efforts are therefore required to improve the genetic diagnosis of psychiatric disorders, as well as their clinical phenotyping and ultimately the design of innovative therapeutic strategies.

## Conflict of Interest Statement

The authors declare that the research was conducted in the absence of any commercial or financial relationships that could be construed as a potential conflict of interest.
